# Evaluation of Renal Fibrosis Using Magnetization Transfer Imaging at 1.5T and 3T in a Porcine Model of Renal Artery Stenosis

**DOI:** 10.3390/jcm12082956

**Published:** 2023-04-19

**Authors:** Deep B. Gandhi, Mina Al Saeedi, James D. Krier, Kai Jiang, James F. Glockner, Lilach O. Lerman

**Affiliations:** 1 Division of Nephrology and Hypertension, Mayo Clinic, 200 First Street SW, Rochester, MN 55905, USA; 2 Department of Diagnostic Radiology, Mayo Clinic, Rochester, MN 55905, USA

**Keywords:** renal artery stenosis, renal fibrosis, magnetization transfer imaging, magnetization transfer ratio

## Abstract

Renal fibrosis is an important marker in the progression of chronic kidney disease, and renal biopsy is the current reference standard for detecting its presence. Currently, non-invasive methods have only been partially successful in detecting renal fibrosis. Magnetization transfer imaging (MTI) allows estimates of renal fibrosis but may vary with scanning conditions. We hypothesized that MTI-derived renal fibrosis would be reproducible at 1.5T and 3T MRI and over time in fibrotic kidneys. Fifteen pigs with unilateral renal artery stenosis (RAS, n = 9) or age-matched sham controls (n = 6) underwent MTI-MRI at both 1.5T and 3T 6 weeks post-surgery and again 4 weeks later. Magnetization transfer ratio (MTR) measurements of fibrosis in both kidneys were compared between 1.5T and 3T, and the reproducibility of MTI at the two timepoints was evaluated at 1.5T and 3T. MTR at 3T with 600 Hz offset frequency successfully distinguished between normal, stenotic, and contralateral kidneys. There was excellent reproducibility of MTI at 1.5T and 3T over the two timepoints and no significant differences between MTR measurements at 1.5T and 3T. Therefore, MTI is a highly reproducible technique which is sensitive to detect changes in fibrotic compared to normal kidneys in the RAS porcine model at 3T.

## 1. Introduction

Chronic kidney disease (CKD) is a progressive condition that results in gradual loss of kidney function over time and has been one of the paramount health problems in the United States as well as worldwide. The CDC estimates that 37 million people in the United States have CKD, although two in five adults with severe CKD might not be aware of their illness [1,2]. Renal fibrosis is a pathological process that is characterized by the accumulation of extracellular matrix proteins and the development of scar tissue. It remains an important pathway in the progression of CKD [3,4,5], where the normal functional kidney tissue is replaced by scar tissue composed of high amounts of collagen and other extracellular matrix components. The accumulation of scar tissue can lead to a reduction in the number of functional nephrons, impairing the kidney’s ability to filter blood and regulate fluid balance. If left untreated, CKD can progress to end-stage renal disease, which can ultimately require renal replacement therapy and might also lead to death. However, studies have shown that if detected early, the progression of renal fibrosis may be slowed down or arrested [6,7]. Therefore, there is a need for an early, safe, and reliable detection of renal fibrosis as well as monitoring its treatment response.

The current reference standard for diagnosing renal fibrosis in CKD and kidney transplant patients is kidney biopsy. However, this approach has several drawbacks, including pain, discomfort, bleeding, sampling bias, prolonged hospitalization and recovery, and the development of fistulas [8,9,10,11]. Due to these limitations, there is a need for non-invasive, possibly imaging-based methods for the safe evaluation of renal fibrosis. There have been many attempts to find novel imaging methods to replace or reduce the frequency of kidney biopsies, but none have proven sufficiently successful. Methods such as contrast-enhanced computed tomography (CT) can determine the presence of renal fibrosis indirectly using measurements of renal blood flow and glomerular filtration rate [12,13,14,15,16]. However, CT uses ionizing radiation and requires the administration of intravenous contrast agents in most cases. These factors render CT difficult to employ clinically for determining the presence of renal fibrosis and longitudinally monitoring its treatment response.

In recent years, magnetic resonance imaging (MRI) has emerged as a promising tool compared to other renal imaging modalities such as ultrasound and CT, as it affords high spatial and temporal resolution without requiring any ionizing radiation or external contrast agents. Several MR-based approaches, such as diffusion-weighted imaging (DWI), magnetic resonance elastography (MRE), and functional renal MRI such as blood oxygen level-dependent (BOLD)-MRI (for renal oxygenation) and arterial spin labeling (ASL)-MRI (for renal perfusion) have been used to evaluate renal fibrosis [4,17,18]. However, each of these techniques alone has not been sufficiently consistent in determining the presence of renal fibrosis.

Magnetization transfer imaging (MTI) is an MRI technique that can provide information about tissue composition and microstructure. In traditional MRI, protons in water molecules are excited by radiofrequency pulses and produce a signal that is used to create an image of the body’s internal structures. MTI adds an additional step, where a second radiofrequency pulse is used to selectively saturate protons in specific macromolecules, such as proteins and collagen. The energy from these protons is then transferred to the surrounding water molecules, reducing the signal intensity in the image. By measuring the degree of magnetization transfer, known as the magnetization transfer ratio (MTR), MTI can provide inferences about the amount and distribution of these specialized molecules. In the context of renal fibrosis, MTI can potentially be useful for detecting early-stage fibrosis. This is because fibrotic tissue has a higher density of collagen and other extracellular matrix components, which can alter the magnetization transfer properties of the tissue. By detecting these subtle changes, MTI can provide a sensitive and non-invasive means of monitoring the progression of renal fibrosis over time. Evaluating the molecular underpinnings of renal fibrosis directly independently of other variables may be useful to circumvent the limitations of indirect methods such as MRE or DWI that rely on tissue elasticity or diffusivity and are confounded by renal hemodynamics [19]. Previous studies have shown the feasibility of using MTI in humans and in animal models for detecting renal fibrosis at 3T [19,20,21,22,23,24] but have not applied MTI for renal imaging at 1.5T or tested its reproducibility. Since MTI is semi-quantitative and may vary by scanner and imaging parameters, this study was designed to test the hypothesis that MTI would provide consistent estimates of renal fibrosis at 1.5T and 3T in a porcine model of renal artery stenosis (RAS) which would be reproducible 4 weeks apart.

## 2. Materials and Methods

### 2.1. Study Protocol

This study was approved by the Institutional Animal Care and Use Committee. A total of 15 female domestic pigs were used, including nine pigs with unilateral RAS and six age-matched sham control pigs. To introduce RAS in 9 pigs, intravenous heparin (5000 U) was administered, which was followed by the insertion under fluoroscopic guidance of a 5.0 mm balloon catheter with local irritant wire coils (23-gauge) wrapped around it. The catheter was engaged in the left renal artery through an aortic guide over a 0.014-inch guide wire and positioned in the proximal-middle section of the renal artery. The balloon was inflated once at high pressure (14 atm), which resulted in coil expansion to full balloon diameter and embedment in the vascular wall. After that, the balloon was deflated and removed. Another dose of heparin was then given, and 15 min later, vessel patency and coil location were ascertained under fluoroscopy with selective renal angiography. The coil was chosen for its ability to generate a progressive proliferative response of the vessel wall and consequently luminal obstruction due to RAS. We have previously shown that this intervention leads to the development of renal fibrosis [25,26]. In the remaining pigs, a sham procedure was performed. In vivo MRI studies were performed on all pigs 6 weeks and again 4 weeks after the RAS or sham surgery to evaluate the reproducibility of MTR. The MRI studies at each timepoint were performed at two different field strengths, 1.5T and 3T, to evaluate the effect of scanner field strength on the consistency of renal fibrosis measurements. Finally, the pigs were euthanized following the MRI studies.

### 2.2. MRI Studies

MRI studies were performed on GE Signa HDxt 1.5T and GE Signa HDxt 3.0 T MRI scanners (GE Healthcare, Waukesha, WI, USA). Prior to the MRI scans, all pigs were initially anesthetized by intra-muscular injection of 5 mg/kg telazol and 2 mg/kg xylazine. All pigs then underwent endotracheal intubation to allow for respiratory control during the MRI scanning. Anesthesia was maintained during MRI scanning using 2% isoflurane.

### 2.3. MTI Parameters

All pigs were laid in a supine position, head-first in the MRI scanner, and MTI scanning was performed using a gradient echo (GRE) sequence that included images acquired without MT preparation (M_0_) and MT-weighted images (M_T_). Images at five different levels of the kidney were obtained in the coronal plane with the following imaging parameters for 3T: TR = 300 ms, TE = 4.3 ms, flip angle = 30°, slice thickness = 2.6 mm, field of view = 30 × 30 cm^2^, matrix size = 128 × 128 (reconstructed to 256 × 256) and number of averages = 0.75. Imaging parameters for 1.5T: TR = 300 ms, TE = 5.3 ms, flip angle = 30°, slice thickness = 2.6 mm, field of view = 30 × 30 cm^2^, matrix size = 192 × 128 (reconstructed to 256 × 256) and number of averages = 0.75. MT-weighted images were obtained using Fermi pulses prior to GRE acquisition. MT pulse parameters were as follows: offset frequencies = 600 Hz or 1000 Hz, pulse width = 16 ms, and flip angle = 800°. Two offset frequencies were tested to optimize MTI measurements of fibrosis.

### 2.4. Image Analysis

**Figure 1:**
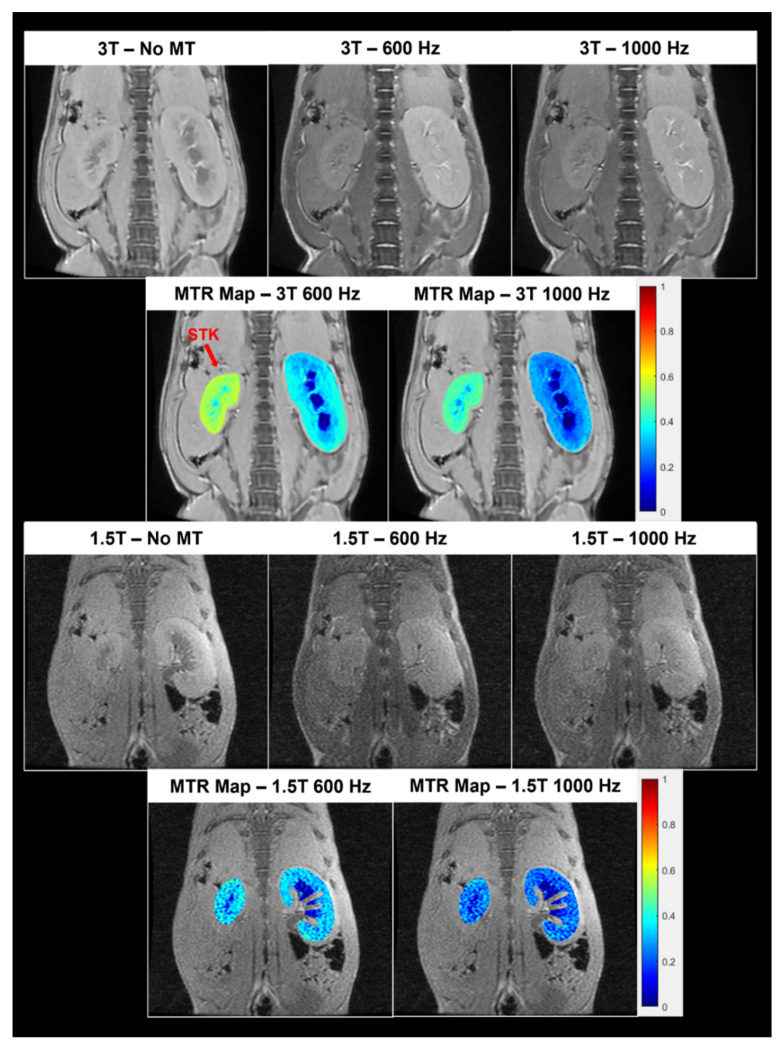
Top. Representative MT images from a pig without MT pulse (i.e., M_0_) and with MT pulse (i.e., M_T_) at 600 or 1000 Hz offset frequencies at 3T, as well as the corresponding MTR maps overlaid on the images. Bottom, M_0_ and M_T_ images from the same pig at 1.5T, and the corresponding MTR maps. MTI at 3T shows better image quality than MTI at 1.5T.

All MR image analysis was performed using in-house custom code developed in MATLAB (MathWorks, Natick, MA, USA). Magnetization transfer ratio (MTR) maps were generated pixel-wise from M_0_ and M_T_ images and calculated using the following formula: $MTR=\frac{\left(M_{0}-M_{T}\right)}{M_{0}}$_._ Figure 1 shows the MT images and corresponding MTR maps at 3T and 1.5T at the two offset frequencies, 600 Hz and 1000 Hz. The cortex and medulla for each kidney were semi-automatically segmented with the help of T2*-weighted anatomical images as shown previously [27] to maintain uniformity across all pigs and prevent bias that may be associated with manually drawn free hand regions of interest (ROIs). Because the contrast in MT images is only moderate, T_1_/T_2_*-weighted anatomical images of the same slices were used to select cortical and medullary ROIs. In T_1_-weighted images, the renal medulla has a longer T_1_, resulting in a lower signal intensity, while in T_2_*-weighted images, it appears darker than the cortex due to lower oxygenation. Therefore, using both T_1_ and T_2_* weighting provides good contrast between the cortex and medulla. A MATLAB-based module was used for semi-automatic ROI selection, which involved excluding the collecting system, thresholding for image segmentation, correcting misclassified pixels in the renal cortex with image opening, and detecting the borders of the cortex and medulla. The threshold and kernel size were both manually adjusted for ROI selection. This method provided satisfactory segmentation of the cortex and medulla, and the selected ROIs were used to calculate their MTRs. MTR values for each cortical and medullary region was normalized by the muscle MTR (obtained by drawing a freehand ROI in the dorsal muscle) for all five slices to adjust for intra and inter-subject B_1_ inhomogeneities. Finally, the mean normalized kidney MTR values were reported for the cortex and medulla in the stenotic (STK), contra-lateral (CLK), and normal kidneys.

### 2.5. Statistical Analysis

All the statistical analyses were completed using MATLAB (MathWorks, Natick, MA, USA). A Wilcoxon rank-sum test was used for comparisons among normal, stenotic, and contralateral kidneys. Paired samples t-test was used for comparisons between first and second timepoints and between MTR obtained at 1.5T and 3T for STK, CLK, and normal kidneys. A *p*-value ≤ 0.05 was considered statistically significant.

## 3. Results

### 3.1. Animal Characteristics

Ten weeks after implantation of the coil, a range of degrees of RAS was induced in the STK (median = 100%; range = 60–100%). These pigs also developed significantly elevated diastolic (131.8 ± 23.7 vs. 86.2 ± 11.8 mmHg, *p* = 0.0004), systolic (166.8 ± 31.4 vs. 123.3 ± 12.8 mmHg, *p* = 0.006), and mean arterial (143.4 ± 25.7 vs. 97.4 ± 11.9 mmHg, *p* = 0.0008) pressures, compared with the normal controls, thereby confirming the functional significance of the stenoses.

### 3.2. MTR Comparison between Normal, STK, and CLK Kidneys

**Figure 2:**
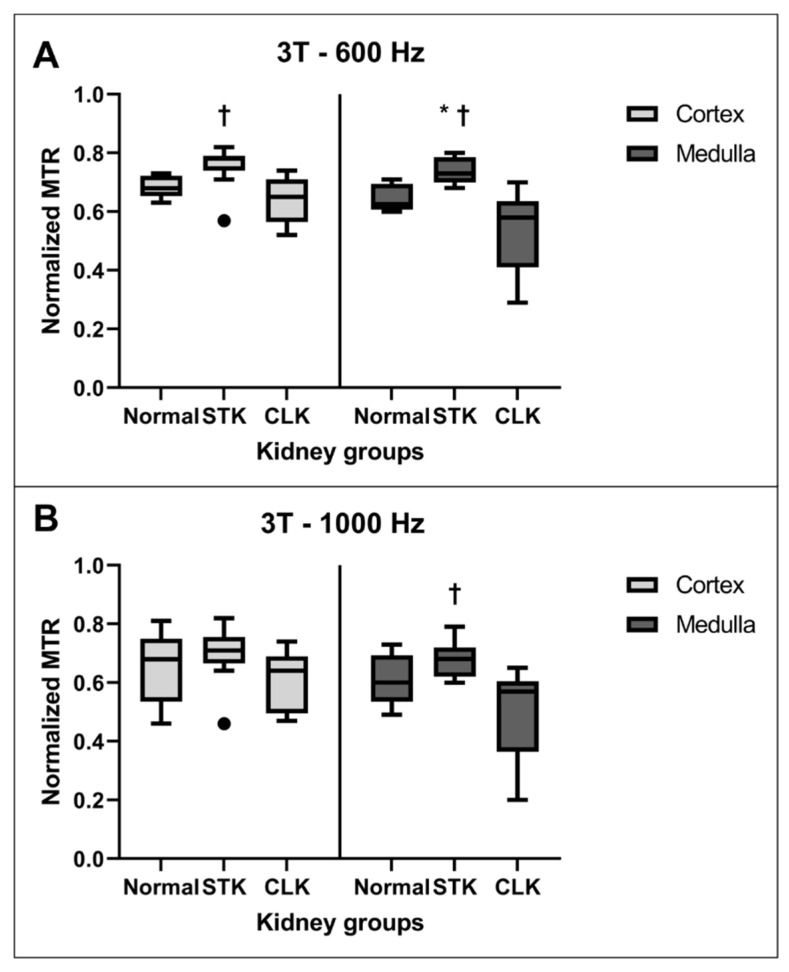
(**A**). Boxplots of normalized MTR values at the first timepoint at 3T with an offset frequency of 600 Hz for normal, stenotic (STK), and contralateral (CLK) kidneys in the cortex (left) and medulla (right). (**B**). Normalized MTR values at 3T with an offset frequency of 1000 Hz for the same kidneys. * *p* ≤ 0.05 vs. normal, † *p* ≤ 0.05 vs. CLK.

Figure 2 shows the normalized MTR values at the first timepoint at 3T in the cortex and medulla for the normal, STK, and CLK kidneys. At an offset frequency of 600 Hz, the STK showed significantly higher MTR values compared with the CLK in both the cortex (*p* = 0.0035) and medulla (*p* = 0.0037) (Panel A). STKs also showed significantly higher medullary MTR than normal kidneys (*p* = 0.014). No other significant differences in the MTR values were observed. At an offset frequency of 1000 Hz, STKs showed significantly higher MTR only compared to CLK medulla (*p* = 0.028, Figure 2B).

**Figure 3:**
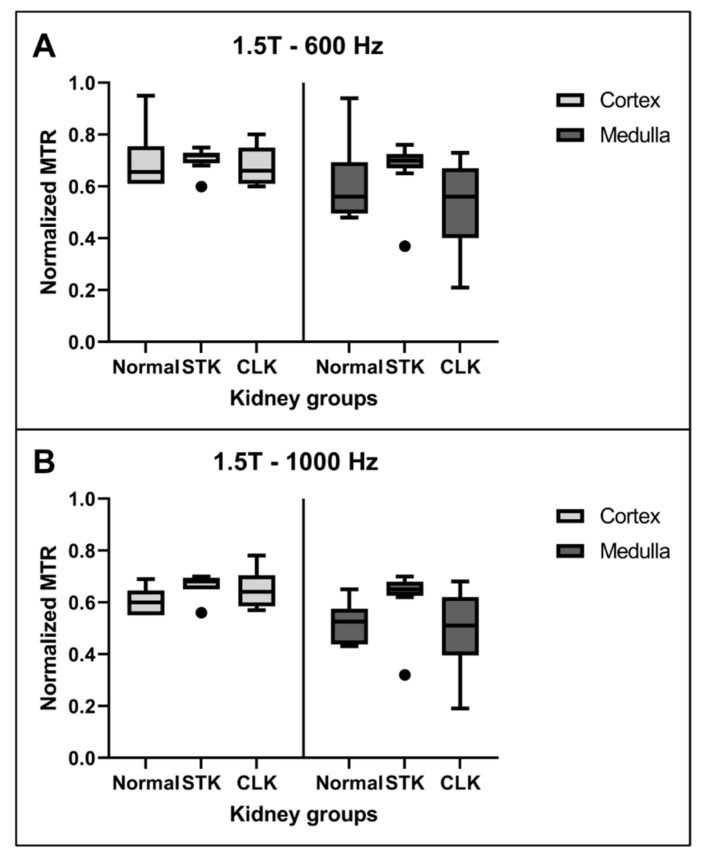
Normalized MTR values at the first timepoint at 1.5T with an offset frequency of 600 Hz (**A**) or 1000 Hz (**B**) for normal, stenotic (STK), and contralateral (CLK) kidneys cortex and medulla. There were no significant differences among any regions or frequencies.

Figure 3 shows the corresponding normalized MTR values at the first timepoint at 1.5T in the cortex and medulla at an offset frequency of 600 Hz or 1000 Hz. No significant difference in MTR values was observed among any of the groups for either offset frequency.

### 3.3. MTR Comparison between 3T and 1.5T

Table 1 shows the MTR values for the different groups and kidney regions at 3T and 1.5T. There was no significant difference between the MTR values obtained at 3T and 1.5T for any of the kidney groups in the cortex and medulla (all *p* > 0.05).

### 3.4. MTR Reproducibility

**Figure 4:**
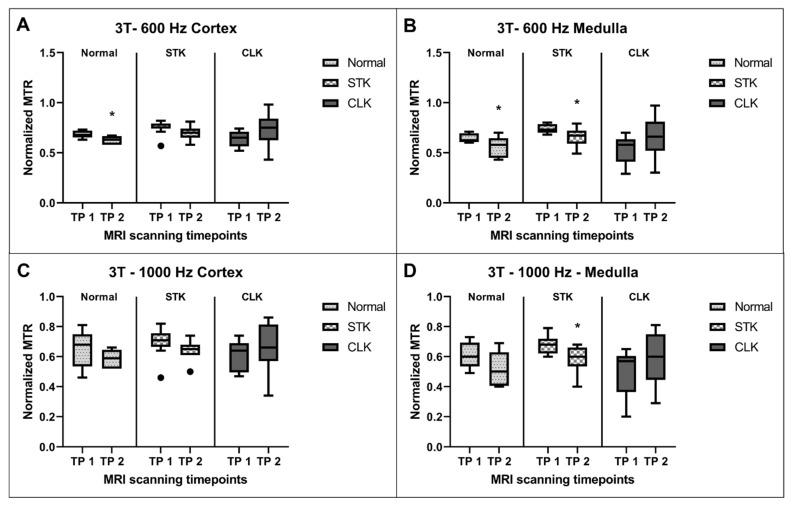
Normalized MTR values compared between the two timepoints (TP) for the normal, stenotic, and contralateral kidneys at 3T at 600 Hz offset frequency in the cortex (**A**) and medulla (**B**) and at 1000 Hz offset frequency in the cortex (**C**) and medulla (**D**). * *p* ≤ 0.05 vs. first TP for the same kidney region.

A comparison of MTR values between the two timepoints for normal kidneys, STK, and CLK at 3T at 600 Hz offset frequency showed significantly lower MTR values at the second timepoint in the normal kidneys in the cortex (*p* = 0.008) as well as medulla (*p* = 0.044) (Figure 4). Similar results were observed for the STK medulla (*p* = 0.026). There was no difference in the MTR values across the two timepoints for any of the remaining comparisons.

At 1000 Hz offset frequency, the MTR value was significantly lower in the second compared to the first timepoint in the STK medulla (*p* = 0.033), but this was not the case in any other region.

**Figure 5:**
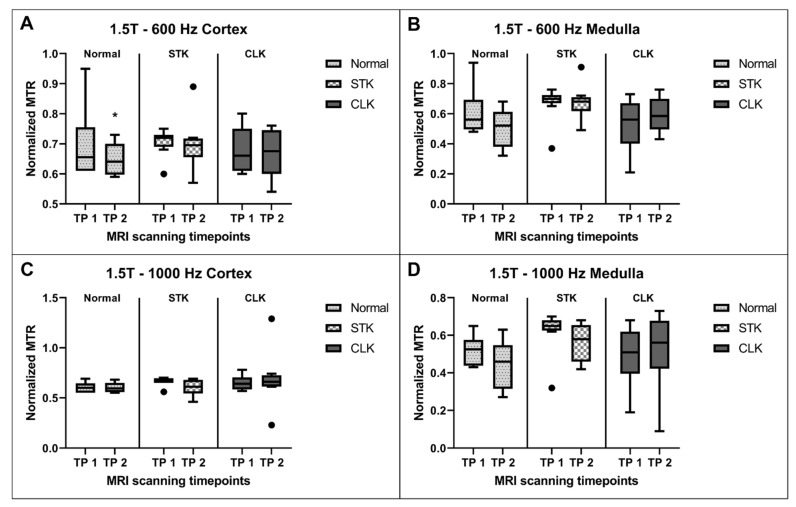
Normalized MTR values compared between the two timepoints (TP) for the normal, stenotic, and contralateral kidneys at 1.5T at 600 Hz offset frequency in the cortex (**A**) and medulla (**B**) and at 1000 Hz offset frequency in the cortex (**C**) and medulla (**D**). * *p* ≤ 0.05 vs. first TP for the same kidney region.

Similarly, Figure 5 shows the comparison between MTR values across the two timepoints at 1.5T for offset frequencies of 600 Hz and 1000 Hz in the cortex and medulla. Cortical MTR was significantly lower at the second timepoint in the normal kidneys at 600 Hz offset frequency. There was no significant difference within any other groups. Notably, MTR values showed greater variability at 1.5T compared to 3T at both frequencies.

## 4. Discussion

This study shows that the magnetization transfer imaging-based MTR at 3T and offset frequency of 600 Hz can differentiate between normal and fibrotic kidneys, as evidenced by significant differences in MTR values detected between the STK and normal kidneys as well as between the STK and CLK both in the cortex and medulla regions. Furthermore, this study demonstrates excellent reproducibility of MTR obtained using both 1.5T and 3T. Additionally, this study also shows no significant differences in the normalized MTR values obtained using 1.5T and 3T, arguing against the field-strength dependence of MTI. These observations support the use of MTI-derived MTR to assess regional kidney fibrosis in vivo in ischemic kidneys.

Advances in emerging MR-based quantitative imaging techniques have great potential in evaluating renal fibrosis, which is an important biomarker of the progression of CKD. These techniques can potentially address limitations of the current reference standard as well as several proposed techniques for the evaluation of renal fibrosis. In the kidney, MTI can be a powerful tool for detecting early-stage fibrosis, which is difficult to reveal using traditional MRI techniques such as T_1_ or T_2_-weighted imaging, MRE, or DWI. MTI has several advantages over other imaging techniques for detecting renal fibrosis. We have previously shown that MRE and DWI, indirect methods to assess kidney fibrosis that rely on tissue elasticity or diffusivity, vary with changes in renal blood flow and perfusion pressure, whereas MTI remains more stable [19]. MTI also does not require the use of complex software packages or licenses for image processing and analysis and can be performed on standard MRI equipment. This makes it a safe and cost-effective option for monitoring disease progression and treatment response over time. In addition, MTI can inform about the specific regions of the tissue that are affected by fibrosis, which may be useful for developing targeted therapies. It also has the advantage of providing quantitative information about the presence of macromolecules in tissues with MTR and can be sensitive to microstructural changes in tissues. In the current study, the efficacy of MTI to distinguish between normal and fibrotic kidneys was assessed in a swine model of RAS as was its dependence on field strengths (1.5T and 3T) and offset frequencies (600 Hz and 1000 Hz). Additionally, the reproducibility of MTI at each field strength and offset frequency over time was also tested.

To the best of our knowledge, this is the first study evaluating the reproducibility of MTI in ischemic kidneys as well as between 1.5T and 3T field strengths. Previous studies have demonstrated the feasibility of MTI in kidneys at 3T [19,27,28,29]. In the present study, MTI showed good reproducibility at 3T, but we observed significant differences over time in the MTR values in the normal kidney cortex and medulla at 600 Hz and in the STK medulla at both 600 Hz and 1000 Hz. A fall in the MTR values in normal kidneys could be due to the growth of our juvenile pigs over the 4-week observation. Previous studies have shown a decline in renal expression of α-smooth muscle actin and vimentin, which characterize collagen-producing myofibroblasts during kidney maturation [30]. We have also found a decrease in renal vascular resistance during the maturation of juvenile pigs [31]. Similar to 3T, MTI at 1.5T showed excellent reproducibility except for the significant fall in MTR normal kidneys at 1.5T at 600 Hz offset frequency. A more modest fall in STK MTR between the two timepoints compared to normal kidneys might have been offset due to the progression of renal fibrosis.

Interestingly, we found no significant differences in the average normalized MTR values between 1.5T and 3T, suggesting independence of MTR from MRI field strength. The major reason for non-differential MTR values at 1.5T and 3T may be the use of normalization between renal MTR and dorsal muscle MTR. Although the purpose of using this normalized MTR is to account for B1 inhomogeneity, it may also minimize the dependence of MTI on field strength. Yet, MTR at 1.5T was not able to differentiate between normal kidneys, STK and CLK; whereas MTR at 3T, specifically at 600 Hz, was able to distinguish between normal and fibrotic kidneys. This is in agreement with the previous studies by Jiang et al. in a swine model of atherosclerotic RAS as well as in a murine model of RAS [21,27]. Notably, we observed a noticeable difference in image quality using MTI between 1.5T and 3T (Figure 1). The lower overall signal-to-noise ratio and spatial resolution of 1.5T compared to 3T MRI [32,33] could lead to reduced sensitivity as well as greater variability of MTI at 1.5T. It should also be noted that the MT effect is stronger at higher field strengths due to longer relaxation times [34], which could explain the higher sensitivity of MT at 3T to detect fibrosis.

Overall, these results show that the MTI technique can potentially be applied in clinical settings, where a higher MTR, compared against a baseline or reference MTR, can be indicative of renal fibrosis. MTI, incorporated within a multi-parametric renal imaging protocol may therefore be a useful clinical tool in determining the presence of renal fibrosis. MTI successfully detected renal fibrosis in our model of unilateral RAS but can be applied in bilateral models as well to detect fibrosis once a range of normal values is determined in healthy individuals as a reference standard.

This study has a few limitations. Firstly, our sample was rather small. Secondly, the 4-week gap between the MRI scans to test reproducibility could have led to changes in the renal physiology (e.g., maturation) and pathophysiology in RAS pigs. Third, the apparent lower MTR compared to normal and its increase over time in the CLK medulla were not statistically significant but could speculatively result from the development of pressure natriuresis and subsequently fibrosis, respectively, secondary to renovascular hypertension. Fourth, diminished cortico-medullary differentiation due to reduced SNR and spatial resolution at 1.5T could have affected the regions of interest selected for MTR measurements. Fifth, although we found little dependence on field strength, MTR remains a semi-quantitative metric to measure macromolecular content, as it depends on several scanning parameters including the duration and frequency of MT pulses and does not take into account the contribution of B_0_ and B_1_ field inhomogeneities. This limitation may potentially be addressed using quantitative magnetization transfer imaging, which provides a more direct assessment of the macromolecular content in kidneys with the measurement of the bound pool fraction. Sixth, future studies will need to compare MTR measurements to histology-based renal fibrosis, which could provide a direct evaluation of the efficacy of MTI-based MTR measurements in determining the presence of renal fibrosis. Finally, fibrosis may also be patchy, which may require several slices to be acquired through the kidney in order to detect fibrotic foci. While we have previously demonstrated the spatial agreement between MTR and histology at 16.4T MRI [21], further studies are needed to determine this capability at 1.5T or 3T.

## 5. Conclusions

In conclusion, this study demonstrated the feasibility and excellent temporal reproducibility of MTI at 1.5T and 3T at 600 and 1000 Hz offset frequencies. MTI at 3T with an offset frequency of 600 Hz was the most sensitive in distinguishing changes between normal, stenotic, and contralateral kidneys in the cortex and medulla. Overall, MTI is a promising imaging technique for detecting and monitoring renal fibrosis and may help improve the diagnosis, management, and treatment of CKD. Future studies with larger sample sizes, along with quantitative magnetization transfer techniques for non-invasive evaluation of renal fibrosis, are warranted to confirm our findings.

## Figures and Tables

**Table 1:** Magnetization transfer ratios (MTR) in normal, stenotic, and contralateral kidneys at 1.5T and 3T and 600 Hz and 1000 Hz offset frequencies.

Region	Kidneys	3T—600 Hz	1.5T—600 Hz	3T—1000 Hz	1.5T—1000 Hz
Cortex	Normal	0.68 ± 0.04	0.69 ± 0.13	0.65 ± 0.13	0.60 ± 0.05
	Stenotic	0.75 ± 0.08	0.71 ± 0.04	0.70 ± 0.10	0.67 ± 0.04
	Contralateral	0.64 ± 0.08	0.69 ± 0.07	0.61 ± 0.10	0.66 ± 0.07
Medulla	Normal	0.64 ± 0.04	0.61 ± 0.17	0.61 ± 0.09	0.52 ± 0.08
	Stenotic	0.74 ± 0.04	0.67 ± 0.12	0.68 ± 0.06	0.62 ± 0.11
	Contralateral	0.53 ± 0.15	0.52 ± 0.17	0.50 ± 0.17	0.50 ± 0.16

Data are means ± standard deviations.

## Data Availability

The data presented in this study are available on request from the corresponding author.
